# Study on Low Thermal-Conductivity of PVDF@SiAG/PET Membranes for Direct Contact Membrane Distillation Application

**DOI:** 10.3390/membranes13090773

**Published:** 2023-08-31

**Authors:** Jun Xiang, Sitong Wang, Nailin Chen, Xintao Wen, Guiying Tian, Lei Zhang, Penggao Cheng, Jianping Zhang, Na Tang

**Affiliations:** 1 Tianjin Key Laboratory of Brine Chemical Engineering and Resource Eco-Utilization, College of Chemical Engineering and Material Science, Tianjin University of Science and Technology (TUST), 13th Avenue 29, TEDA, Tianjin 300457, China; jxiang@tust.edu.cn (J.X.); 22811902@mail.tust.edu.cn (S.W.); chennl1995@163.com (N.C.); 20032216@mail.tust.edu.cn (X.W.); guiying.tian@tust.edu.cn (G.T.); leizhang@tust.edu.cn (L.Z.); cpg@tust.edu.cn (P.C.); zhangjianping@tust.edu.cn (J.Z.); 2 State Key Laboratory of Biobased Fiber Manufacturing Technology, Tianjin University of Science and Technology, 13th Avenue 29, TEDA, Tianjin 300457, China

**Keywords:** non-solvent induce phase separation, hydrophobic/hydrophilic membrane, silica aerogel, low thermal-conductivity, membranes distillation

## Abstract

In order to enhance the separation performance and reduce the heat loss of transmembrane for membrane distillation, the thermal efficiency and hydrophobicity of the membrane distillation need to be simultaneously enhanced. In this work, a polyvinylidene difluoride/polyethylene glycol terephthalate (PVDF/PET) hydrophobic/hydrophilic membrane has been prepared by non-solvent phase induction method. Nanosized silica aerogel (SiAG) with high porosity has been added to the composite membranes. The modifying effects and operating conditions on permeate flux and thermal efficiency in direct contact membrane distillation (DCMD) are investigated. Furthermore, the latent heat of vaporization and the heat transfer across the membranes have been compared for SiAG addition, which indicates that the composite PVDF@SiAG/PET membranes demonstrate a great potential for distillation-separation application due to their high heat efficiency.

## 1. Introduction

Fresh water is essential to life everywhere, but the natural water systems are being disrupted due to domestic activity and industrial overuse. Thus, the world is facing the stress of limited water resources coupled with inadequate infrastructure to protect water [1]. Therefore, many sewage purification technologies have been developed. Among them, membrane distillation (MD) is a high-efficiency membrane separation technology using the vapor pressure difference of the transmembrane as a driving force [2]. In the MD unit, a porous non-selective hydrophobic membrane acts as a physical barrier, which can separate water vapor from the warm aqueous solution. In general, the MD is a nonisothermal separating process that combines thermal evaporation and membrane separation, offering the low-cost advantages of low operating pressure and temperature [3]. Therefore, MD technology has been widely applied in seawater desalination, ultrapure water preparation, wastewater treatment, the separation of azeotropic mixtures, etc. [4]. The performance of MD depends on the membrane properties and operating conditions. Moreover, high-performance membranes for MD should satisfy the following characteristics: (1) low thermal conductivity to reduce heat loss across the membrane; (2) low transport resistance to diffusion of vapor molecules to increase permeate flux; (3) high mechanical durability and structural stability; (4) high liquid entry pressure to prevent wetting of the transmembrane [5]. Therefore, special membrane structures for the MD application need to be continuously optimized.

In order to improve the MD performance, the conventional method is to prepare a thinner membrane to achieve lower mass transfer resistance and higher permeation flux. However, the membrane with low mechanical strength fails to withstand the liquid pressure between the feed side and the permeable side. Further, the thinner membrane also increases the heat transfer loss of the membrane, and the reduced temperature difference of the membrane results in a decrease in driving force and permeable flux. In order to solve the dilemma of membrane thickness, constructing the hydrophobic/hydrophilic composite membrane is an effective modifying method since the thinner hydrophobic layer and the hydrophilic layer across the MD membrane can improve the permeable flux by shortening the transmission path of water vapor, as well as reducing mass transfer resistance. Thus, the combination of hydrophobic layer and hydrophilic layer on the same MD membrane can diminish the conductive heat loss of the transmembrane [6].

So far, the most commonly used hydrophobic polymers in the MD membrane are polytetrafluoroethylene (PTFE), polypropylene (PP), and polyvinylidene fluoride (PVDF). Among them, PVDF membranes possess stable chemical properties and offer ultra-high hydrophobicity [7]. However, the thermal conductivity of PVDF is too high (0.1652–0.1848 W∙mK^−1^) to repress the heat loss of the transmembrane. The introduction of inorganic microparticles with low thermal conductivity into the composite membrane is expected to reduce heat loss in the MD application. For example, SiO_2_ aerogel is widely considered as a heat-insulating material with excellent thermal insulation, which possesses low thermal conductivity of 0.012–0.030 W∙mK^−1^ and high hydrophobicity of ~150° contact angle [8]. In 2014, Li et al. [9] reduced the thermal conductivity of polysulfone/polyvinylidene difluoride (PSF/PVDF) membranes by using SiO_2_ aerogel for the DCMD process.

Additionally, the selection of the hydrophilic sub-layer (base membrane) plays a significant role in determining the permeable flux in MD applications. It is known that the nuclear track membrane, a novel material with a uniform pore size distribution and straight pore structure, shows promise as a high-precision filtration membrane in sewage treatment. Furthermore, the polyethylene terephthalate (PET) nuclear track membrane, a semi-crystalline hydrophobic membrane, presents excellent mechanical strength, regular pores, and chemical resistance. Thus, it is very appealing to integrate hydrophobic PET nuclear track membrane and hydrophilic porous PVDF membrane for MD application because such PVDF/PET hydrophobic/hydrophilic membranes can combine a low mass transfer resistance by shortening path length of the water vapor transport through the hydrophobic thin top-layer and a low conductive heat loss through the membrane obtained by using thicker hydrophilic sub-layer of the transmembrane [10].

With the aim to enhance overall permeability and thermal insulation properties, the hydrophobic and hydrophilic layers in the MD application warrant further investigation. Thus, the hydrophobic/hydrophilic PVDF/PET membrane by using non-solvent-induced phase method (NIPS) is prepared in this work [11]. In addition, we conduct an in-depth examination of the varying SiO_2_ aerogel contents within the PVDF@SiAG/PET composite membrane. The corresponding porous structure and separation performance of the composite membrane are also characterized concerning its direct contact membrane distillation (DCMD) application [12]. Herein, some DCMD parameters, such as permeate flux, retention rate, heat transfer, and thermal efficiency, are also compared for separation performance.

## 2. Materials and Methods

### 2.1. Materials and Reagents

Polyvinylidene fluoride powder (PVDF, FR904, Shanghai 3F New Materials Co., Ltd., Shanghai, China), PET nuclear-track membrane (water contact angle of 60°, Liaoning Dongdaihe Tianzhirun Technology Co., Ltd, Suizhong, China, see Figure S1), nanosized SiO_2_ aerogel powder (abbreviated as SiAG, Langfang Zall Thermal Insulation Material Co., Ltd., Langfang, China), anhydrous lithium chloride (LiCl, analytical reagent, Shanghai Aladdin Biochemical Technology Co., Ltd., Shanghai, China), anhydrous ethanol (purity > 99.7%, Tianjin Jiangtian Chemical Technology Co., Ltd., Tianjin, China), sodium chloride (NaCl, analytical reagent, Shanghai Aladdin Biochemical Technology Co., Ltd., Shanghai, China) are commercially available. N, N-dimethylacetamide (DMA, analytical reagent), and acetone (AC analytical reagent) are from Tianjin Damao Chemical Reagent Factory. These commercial chemicals were directly used without additional purification. Deionized (DI) water was purified through Dow XLE-2521 membranes in our lab (total conductivity < 2 μS·cm^−1^).

### 2.2. Preparation of the Hydrophobic/Hydrophilic Membranes

**Figure 1:**
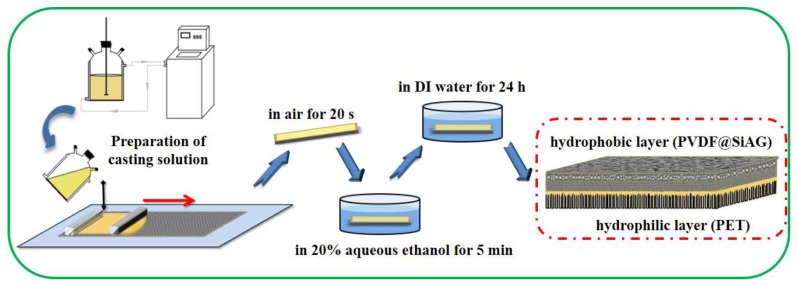
Schematic preparation of PVDF@SiAG/PET hydrophobic/hydrophilic membranes.

Figure 1 illustrates the PVDF@SiAG/PET membranes with low thermal conductivity were prepared using the NIPS method. The casting solution was prepared by mixing LiCl, AC, and DMA for 1 h, following which various mass ratios of SiAG, as indicated in Table 1, were incorporated. Thereafter, the PVDF powder was added and stirred for 2 h in a water bath at 60 °C to form a uniform casting solution. After standing for 12 h at room temperature for defoaming, this solution was cast onto the surface of PET nuclear-track membranes (as the sub-layer) with a wet-thickness of 100 μm. After being exposed to air for 20 s, the as-prepared composite membrane was immersed in 20% aqueous ethanol solution for 5 min, and then transferred into DI water for 24 h immersion. Finally, the PVDF@SiAG/PET hydrophobic/hydrophilic membranes were acquired. Herein, these membranes were abbreviated as M-X, where X hereinafter refers to the corresponding mass ratio of SiAG to PVDF in the composite membranes. As references, the PVDF membranes without SiAG on PET sub-layer were also prepared [13].

### 2.3. Structural Characterization

A scanning electron microscope (SEM, Phenom Pure, Netherlands Phenom-World Ltd., Eindhoven, Noord-Brabant, The Netherlands) was used to observe the top surface of the membranes. The cross-sectional surface of the membranes was prepared by liquid nitrogen fracturing. Then, these samples were covered with sputtered gold using an Ion Sputtering device (SBC-12, KYKY Technology Co., Ltd., Beijing, China) for cross-sectional observation. A Tecnai G2 F-20 analyzer (FEI Company, Hillsboro, OR, USA) was used for energy spectrum analysis carried out to explore the distribution of Si distribution on the membrane surface.

The hydrophobicity of the membranes was characterized by IL 4200 Contact Angle Goniometer with Drop shape analysis (DSA100, KRÜSS Scientific, Hamburg, Germany). The average pore size, pore size distribution, and air permeability of the membranes were characterized by a Capillary Flow Pyrometry (POROLUX 1000, Porometer Instrument Co., Ltd., Leuven, Vlaams-Brabant, Belgium). The porosity of the membranes was determined by the gravimetric method. The dry membrane sample (*m_d_*) was cut into a circular with a diameter of 25 mm, and its average thickness (*h*) was measured using a spiral micrometer (Shanghai Tool Works Co., Ltd., Shanghai, China). Then, the sample was immersed in the Porefil^®^ infiltration solution for 12 h. After absorbing the residual infiltration solution on the surface using filter paper, the weight of the wet membranes (*m_w_*) was obtained. Then, the porosity (*ω*, %) was calculated according to Equation (1):

(1) $$\omega =\frac{m_{w}-m_{d}}{Ah\rho }\times 100\%$$

where *ρ* is the profile density of the infiltration solution (1.86 g·cm^−3^); *A* is the membrane area (cm^2^).

### 2.4. Thermal Conductivity Test

The thermal conductivity of the membranes was measured using a thermal constant analyzer (Hot Disk TPS2500, Kegonas, Uppsala, Sweden). The relationship between porosity and thermal conductivity is shown in Equation (2):

(2) $$\lambda =\lambda_{v}\varepsilon +\lambda_{m}\left(1-\varepsilon \right)$$

where *λ* (W·mK^−1^) is the total thermal conductivity of the membranes; *λ_v_* and *λ_m_* are the thermal conductivity from the void (air or water vapor) and the bulk materials in the membranes, respectively. Based on Equation (3), *λ_m_* is calculated by the volume fractions of PVDF to SiAG:

(3) $$\lambda_{m}=\frac{1}{1+\frac{P_{SiAG}}{P_{PVDF}}}\left(\lambda_{PVDF}-\lambda_{SiAG}\right)+\lambda_{SiAG}$$

where *λ_PVDF_* and *λ_SiAG_* are the thermal conductivity of PVDF polymer and SiAG particles, respectively. *P_PVDF_* and *P_SiAG_* are the volume fractions of PVDF polymer and SiAG particles in the membranes, respectively.

### 2.5. Batch Test of the DCMD

**Figure 2:**
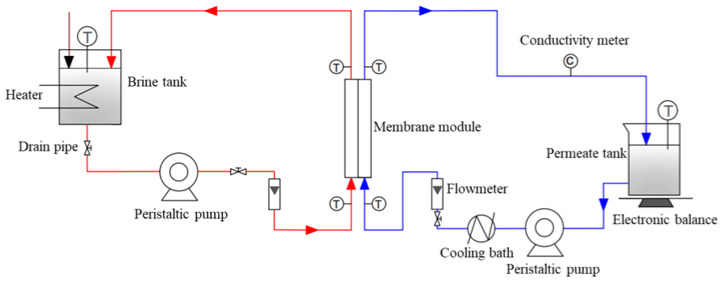
Schematic illustration of the experimental used for DCMD test.

The separation performance of DCMD test was investigated by using a 3.5 wt.% NaCl solution as feed liquid. Figure 2 illustrates the experimental instrument for the DCMD test. The feed liquid in the brine tank was heated up to 65 °C, and the permeation temperature was controlled to 20 °C. The peristaltic pump was running with a feed flow of 35 L·h^−1^. The feed liquid from the brine tank went through the membrane’s module with an effective membrane area of 72 cm^2^. In the DCMD test, the water vapor in the feed liquid was transferred through the pores of the membranes and then was cooled down and collected in the permeate tank [14]. At the same time, the permeable liquid mass with the corresponding total dissolved solid (TDS) was measured at regular intervals. The permeation flux (*J_w_*, kg·m^−2^·h^−1^) was calculated based on Equation (4):

(4) $$J_{w}=\frac{∆w}{A\cdot t}$$

where ∆*w* is the mass of the permeate (kg); *A* is the effective membrane area (m^2^); ∆*t* is the distillate collection time (h).

Rejection rate (%) of NaCl solution was calculated based on Equation (5):

(5) $$R=\frac{C_{f}-C_{p}}{C_{f}}\times 100\%$$

where *C_f_* is the feed liquid concentration (mg·L^−1^); *C_p_* is the permeate concentration (mg·L^−1^).

Latent heat of vaporization (*H_L_*, W·m^−2^) in the heat transfer process of the DCMD was calculated based on Equation (6):

(6) $$H_{L}=\frac{J_{w}r}{3.6\times 10^{3}}$$

where *J_w_* is the permeation flux of water (kg·m^−2^·h^−1^); *r* = 2.3455 × 106 J·kg^−1^ is the heat of water vaporization of water (J·kg^−1^).

Thermal conductivity (*H_C_*, W·m^−2^) of the membranes was calculated *f* based on Equation (7):

(7) $$H_{C}=\frac{\lambda }{l}\left(\frac{T_{fi}+T_{fo}}{2}-\frac{T_{pi}+T_{po}}{2}\right)$$

where *λ* is the thermal conductivity of the membranes (W·m^−1^·K^−1^); *l* is the membrane thickness (m); *T_fi_* and *T_fo_* are the inlet and outlet temperatures of the liquid feed, respectively, *T_pi_* and *T_po_* are the inlet and outlet temperatures of the permeate, respectively. The temperature polarization was ignored in the calculation of *H_C_*. The thermal efficiency (*η*, %) in the DCMD test was calculated based on Equation (8):

(8) $$\eta =\frac{H_{L}}{H_{L}+H_{C}}\times 100\%$$

## 3. Results and Discussion

### 3.1. Characterization of the PVDF@SiAG/PET Membranes

**Figure 3:**
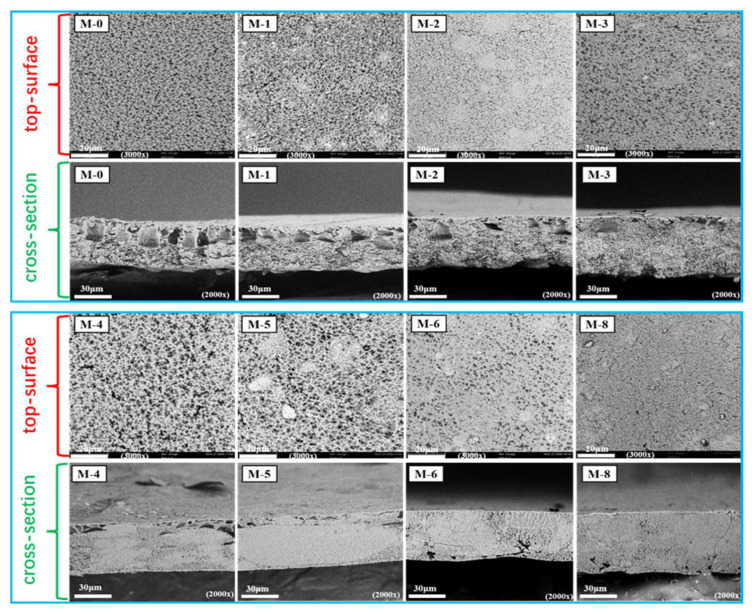
SEM images of the top surface and cross-section of PVDF@SiAG/PET membranes with different R_SiAG_.

Figure 3 presents the top-surficial SEM images of the PVDF@SiAG/PET composite membrane. The M-0 displays uniformly distributed pores over the top surface. After adding SiAG, the top-surface SEM images of the membranes exhibit lumpy particles with low homogeneity. Furthermore, the distribution of SiAG particles of the M-1, M-2, M-3, and M-4 is relatively homogeneous with low added amount. However, the SiAG particles on the surface of the M-5, M-6, and M-8 are clumped together. Notably, M-0 without SiAG displays typical finger-like pores throughout the spongy membrane, which can also be observed in the cross-sectional image. As more SiAG is added, the finger-like pores gradually disappear. When the R_SiAG_ exceeds 0.5, the sponge-like membrane becomes much denser with the vanishing finger-like pores. Thus, the adding amount of SiAG nanoparticles determines the change of the membrane’s structure. This is because SiAG nanoparticles served as the nucleating agent during the NIPS process, which speeded up the phase transition by increasing the crystallization rate of PVDF polymer [15].

**Figure 4:**
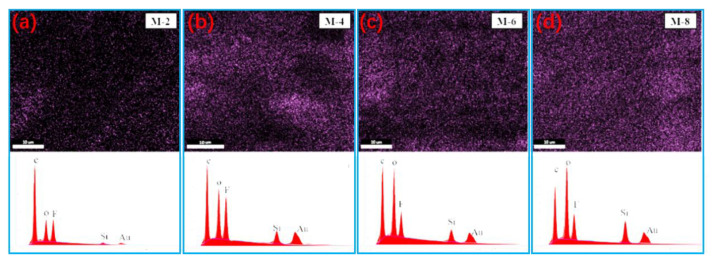
(**a**–**d**) are the energy spectral analysis of Si elements in M-2, M-4, M-6 and M-8 PVDF@SiAG/PET films, respectively.

Figure 4a–d represents the energy spectrum analysis and electron microscope images of M-2, M-4, M-6, and M-8, respectively. The electron microscope images show the variations in different Si atoms in the film. It is readily observable that SIAG particles are uniformly distributed in the film matrix, and the silicon content in M-2, M-4, M-6, and M-8 is 3.26%, 8.78%, 10.31%, and 14.62%, respectively, indicating that the silicon content on the modified film surface increases with the increase of SIAG content, and the distribution in the film is relatively uniform, indicating good dispersion. However, the excessive SiAG nanoparticles are trapped in the PVDF polymer matrix, resulting in a decrease in the average pore size, as seen in Figure 3. As a result, the inhomogeneous structure in the PVDF top layer would deteriorate the structural stability and vapor permeability [16].

To study the thermal stability of the composite membrane, a comparative thermogravimetric analysis of the PVDF/SiAG composite membrane and the bare PVDF membrane is presented in Figure S2. The thermogravimetric curves demonstrate that the mass of the bare PVDF membrane begins to decrease when the temperature is above 366 °C, whereas the mass of the PVDF/SiAG membrane without PES begins to see a decrease at temperatures exceeding 459 °C, indicating that the addition of the inorganic SiAG aerogel is beneficial to enhancing the thermal stability of the PVDF membrane [17].

**Figure 5:**
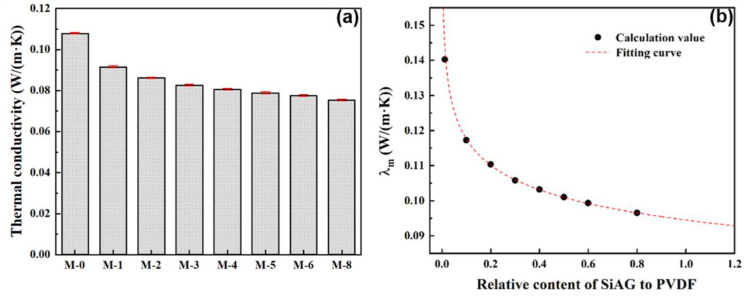
Effect of varying SiAG content on the thermal conductivity (**a**), and the corresponding fitting analysis of the *λ_m_*-R_SiAG_ in the PVDF@SiAG/PET membranes (**b**).

In this study, the thermal conductivity of the PVDF@SiAG/PET composite membranes was measured using a thermal constant analyzer. As shown in Figure 5a demonstrates that the thermal conductivity of PVDF@SiAG/PET membrane is decreased with increasing R_SiAG_. Thus, M-0 displays the maximum thermal conductivity of 0.1079 W·m^−1^·K^−1^. In contrast, thermal conductivity is 0.0754 W·m^−1^·K^−1^ in the M-0.8, which is reduced by 30.12% when compared with M-0. This indicates that SiAG with an intrinsic thermal conductivity of 0.01 W·m^−1^·K^−1^ can substantially reduce thermal conductivity of the PVDF [18]. Additionally, as depicted in Figure 5b, the calculated *λ_m_* values fit well with the theoretical curves with a correlation coefficient R^2^ = 0.9998. Furthermore, the *λ_m_* decreased by 30.44% when the R_SiAG_ was increased from 0.0 to 0.8. Therefore, the findings show a favorable correlation between the SiAG addition and the decline in thermal conductivity of PVDF@SiAG/PET membranes.

**Figure 6:**
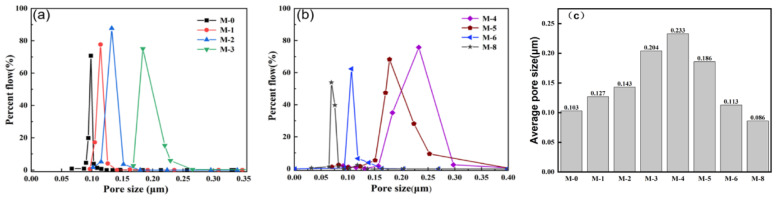
(**a**) Effect of SiAG content on pore size distribution of PVDF@SiAG/PET in M-0, M-1, M-2 and M-3 blended films; (**b**) Effect of SiAG content on pore size distribution of PVDF@SiAG/PET in M-4, M-5, M-6 and M-8 blended films; (**c**) Average pore size of the PVDF@SiAG/PET membranes with varying R_SiAG_.

Furthermore, the pore size distribution in the PVDF@SiAG/PET membrane and the corresponding average pore size are depicted in Figure 6. The average pore size gradually increases from M-0 to M-4, while the average pore size rapidly drops when the R_SiAG_ is above 0.5. As discussed earlier, the addition of SiAG results in the formation of a loose membrane structure.

**Figure 7:**
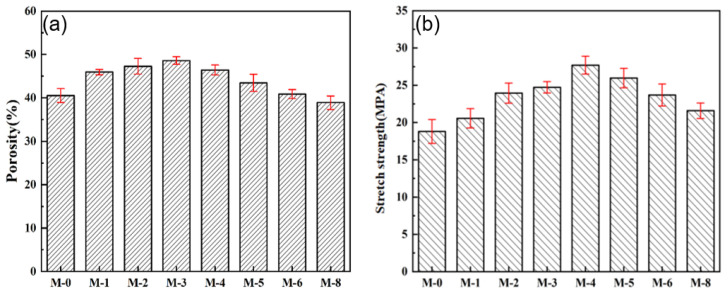
Effect of SiAG content on the porosity (**a**) and tensile strength (**b**) of PVDF@SiAG/PET membranes.

In practical applications, the porous structure and mechanical strength of the membrane are crucial parameters affecting the performance and operational stability of DCMD. Figure 7a shows the effect of SiAG content on membrane porosity. With the increase in SiO_2_ aerogel addition, the porosity of the composite membrane increases from 39.52% for M-0 to 45.60% for M-4 because the low amount of SiAG leads to the formation of loose structure, and thus the internal connectivity of pores is enhanced. However, the excessive SiAG nanoparticles were encapsulated in the PVDF polymer matrix, which resulted in a decrease in the average pore size and porosity, which is consistent with the changes in surface morphology (refer to Figure 3). As further increasing SiAG content, the porosity is reduced from 41.41% for M-5 to 36.60% for M-8. These findings confirm that by adding SiAG nanoparticles, the average pore size and distribution in the PVDF@SiAG/PET membrane can be adjusted [19].

As illustrated in Figure 7b, the tensile strength of PVDF@SiAG/PET membranes initially increased from 19.79 MPa for M-0 to 27.78 MPa for M-4, and then decreased to 21.58 MPa for M-8. This is because SiAG ceramic fillers can be used as strength-enhancing fillers. Moreover, the addition of SiAG can serve as the cross-linking points to physically join the polymeric chains, which can distribute the external load evenly and reduce the possibility of fracture of membrane materials. Therefore, the linked PVDF chains improve mechanical strength. However, the excessive addition of SiAG can form microporous defects during the NIPS process. Additionally, the cracking points are likely to appear at the microporous defects when applying tensile external force, which reduces the tensile strength of PVDF@SiAG/PET membranes. In conclusion, R_SiAG_ = 0.4 is considered the proper adding amount to increase the membrane’s mechanical strength [20].

### 3.2. Effect of PVDF@SiAG/PET Membranes on DCMD

Figure S3 illustrates the effect of SiAG addition on the contact angle of the composite membranes. The contact angle continues to rise from 91.68° for the M-0 to 122.81° for the M-8 as increasing SiAG content. This phenomenon is attributed to the hydrophobic SiAG particles (the static contact angle of modified SiO_2_ is more than 150°) loaded on the PVDF surface [21].

**Figure 8:**
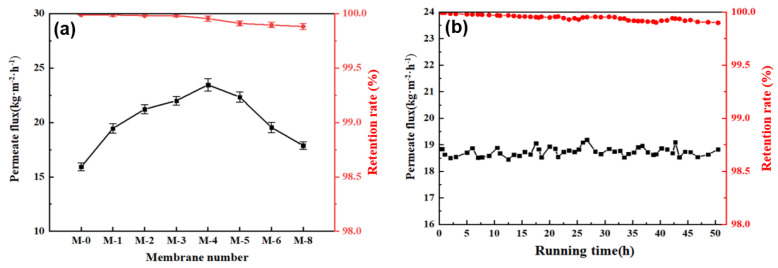
Effect of SiAG content on DCMD performance: (**a**) permeate flux and retention rate of PVDF@SiAG/PET membranes and stable running time of the M−4 membrane (**b**).

In the DCMD test, the flow rate of 35 L/h was set on both the feed side and the permeate side, and the flux temperature of the feed side and permeate side was controlled at 65 °C and 20 °C, respectively. However, the separation performance of the PVDF@SiAG/PET composite membrane was examined in the DCMD process. According to Figure 8a, the maximum permeate flux (23.462 kg·m^−2^·h^−1^) was achieved in the M-4. In contrast to the M-0, the addition of SiAG particles significantly increased the flux, and this is attributed to the addition of silica aerogel, which results in the loosened structure of the composite membrane. However, the excessive addition of SiAG leads to a decrease in the flux, and this is ascribed to the decrease in average pore size and elevated vapor transfer resistance caused by the denser membrane structure.

The stable running time experiment was conducted by using an M-4 membrane, with the experimental conditions set at 70 °C for the feed side and 20 °C for the permeate side flow rates. As shown in Figure 8b, the permeate flux remained stable after approximately 50 h of operation, ranging between 18.514 and 19.136 kg·m^−2^·h^−1^. This finding demonstrates that the addition of hydrophobic low thermal conductivity materials (SiAG) could significantly enhance the permeation rate and separation performance of the PVDF@SiAG/PET composite membranes [22].

Moreover, the retention rates of M-0, M-1, M-2, and M-3 are above 99.99%, whereas the retention rates of M-4, M-5, M-6, M-7, and M-8 range between 99.93% and 99.88%, as shown In Figure 8b. It is most likely a result of the partial loss of SIAG particles induced by the scouring of the feed fluid on the membrane surface. However, the membrane’s spongy structure and pore size are destroyed by the excessive addition of the SiAG. Thus, the M-4 membrane preserves high-performance stability over 50 h, which should be the preferred choice due to its high permeate flux and high retention rate [23].

**Figure 9:**
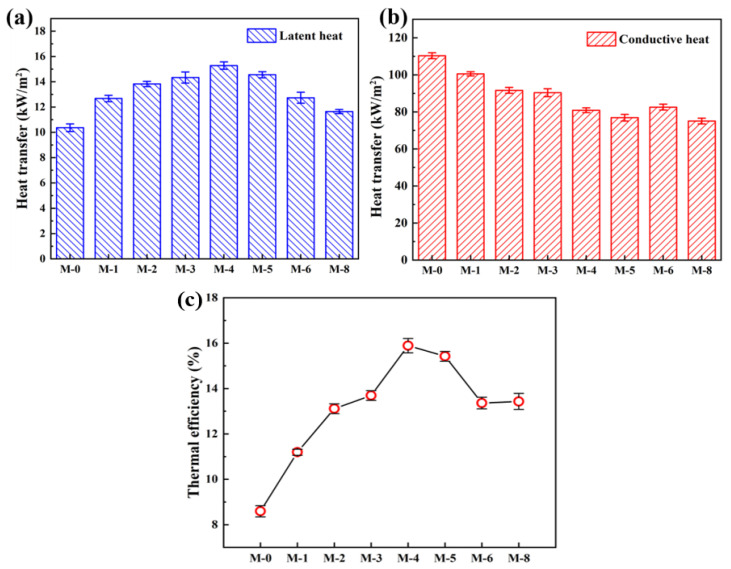
The latent heat of vaporization (**a**), heat transfer (**b**), and thermal efficiency (**c**) of the PVDF@SiAG/PET membranes in the DCMD application.

Figure 9a,b illustrates the latent heat of vaporization and heat transmission across the composite membranes, respectively. M-0 exhibits a latent heat of vaporization of 10.37 kW·m^−2^, while the maximum latent heat of 15.28 kW·m^−2^ is found in the M-4. In Figure 9b, the highest conductive heat of 110.30 kW·m^−2^ is found in the M-0, and the conduction heat gradually decreases with the addition of SiAG. Notably, the PVDF@SiAG/PET membrane with low thermal conductivity can effectively reduce the temperature polarization effect [24] and heat loss during MD test. In addition, the high-temperature gradient across the composite membrane with low thermal conductivity can lead to a greater temperature difference between the permeation side and feed side, which significantly enhances the mass transfer driving force.

As depicted in Figure 9c, the thermal efficiency in the DCMD is defined as the ratio of the latent heat of vaporization to the sum of the latent heat of vaporization and the conduction heat across the membrane. This study reveals that M-0 manifests the lowest thermal efficiency (8.59%), and the SiAG addition greatly enhances the thermal efficiency. Moreover, the M-4 possesses the highest thermal efficiency (15.89%). The findings demonstrate that the latent heat and the conduction heat can determine changes in the thermal efficiency, and this can be attributed to the modification of the PVDF membrane by using SiAG. Significantly, the addition of SiAG enhances the latent heat of vaporization by increasing the average pore size, which in turn increases the permeate flux in the DCMD test. Table 2 summarizes recent research on the performance of low thermal conductivity membranes, and it is found that the model pore size prepared in this study is smaller, and the flux is higher. Therefore, the PVDF@SiAG/PET membrane with R_SiAG_ = 0.4 displays a significant potential for water treatment in terms of enhanced energy saving and thermal efficiency [25].

## 4. Conclusions

In this study, we presented a novel functionalized modification method for PVDF@SiAG/PET composite membranes aiming to reduce the thermal conductivity of MD membrane. The experimental results show that the loosened membrane and improved thermal stability were achieved with the SiAG addition. The proper addition of SiAG (R_SiAG_ = 0.4) results in an increase in pore size and porosity, hydrophobicity, mechanical strength as well as thermal resistance. In the DCMD batch tests, the permeate flux of M-4 reached the maximum value of 23.462 kg·m^−2^·h^−1^, which was 45.5% higher than that of M-0 without adding SiAG, and the DCMD was stably running over 50 h. Therefore, the applied performance of PVDF@SiAG/PET membranes is improved comprehensively by SiAG modification.

## Figures and Tables

**Table 1:** Formulation of casting solution for the PVDF@SiAG/PET membranes.

Samples	PVDF (wt.%)	SiAG(wt.%)	LiCl (wt.%)	Acetone (wt.%)	DMA (wt.%)	R_SiAG_ *
M-0	12.0	0.0	3.0	1.0	84.0	0.0
M-1	12.0	1.2	3.0	1.0	82.8	0.1
M-2	12.0	2.4	3.0	1.0	81.6	0.2
M-3	12.0	3.6	3.0	1.0	80.4	0.3
M-4	6.0	2.4	3.0	1.0	87.6	0.4
M-5	6.0	3.0	3.0	1.0	87.0	0.5
M-6	6.0	3.6	3.0	1.0	86.4	0.6
M-8	6.0	4.8	3.0	1.0	85.2	0.8

* R_SiAG_ is the mass ratio of SiAG to PVDF.

**Table 2:** This article reviews the preparation of low thermal conductivity membranes and the study of membrane distillation performance.

Membrane Sample	Average Pore Size (nm)	Membrane Flux (L/m^2^h)	Rejection Rate (%)	Thermal Conductivity (W·m^−1^·K^−1^)	Ref.	Year
PVDF/SiAG	172	12.50	>99.99%	0.0830	[8]	2020
PVDF/MAF-4	122	27.90	none	0.0458	[26]	2022
PVDF/TBAHP/PS	870	50.00	99.9%	0.0278	[27]	2021
BNNSs/PVDF-co-HFP	720	18.00	99.99%	0.0207	[28]	2020
PVDF	340	9.49	>99%	0.0521	[29]	2018
PVDF/PDMS–SiO_2_	350	12.40	99.9%	0.0620	[9]	2014
PVDF-HNT	440	7.64	100%	0.0597	[30]	2022
PVDF-HFP	390	14.50	99.9%	0.0310	[31]	2021
ZIF-71/PVDF	420	27.10	99.9%	-	[32]	2020
AlFu-MOF-PVDF	297	15.64	>99.9%	0.3561	[33]	2019
PVDF/TNTs	27	92.55	99.9%	-	[34]	2021
PVDF@SiAG/PET	69	23.46	>99.9%	0.0754	this work	2023

## Data Availability

The data presented in this study are available on request from the corresponding author.

## Supplementary Materials

The following supporting information can be downloaded at: https://www.mdpi.com/article/10.3390/membranes13090773/s1, Figure S1: SEM images of PET nuclear-track membranes; Figure S2: Thermal gravimetric diagram of the bare PVDF and the PVDF@SiAG; Figure S3: Dependence of the apparent contact angle on the PVDF@SiAG/PET membranes.
