# Supplementary material for: Study on Low Thermal-Conductivity of PVDF@SiAG/PET Membranes for Direct Contact Membrane Distillation Application
Source: Membranes (Basel). 2023 Aug 31;13(9):773. doi: 10.3390/membranes13090773 (PMC10535353; doi:10.3390/membranes13090773)
Supplement: Supplementary file 1 [file membranes-13-00773-s001.zip › Supporting Information.pdf]

# Supporting Information

## Study on low thermal-conductivity of PVDF@SiAG/PET membranes for direct contact membrane distillation application

Jun Xiang [1,2], Sitong Wang [1], Nailin Chen [1], Xintao Wen [1], Guiying Tian [1,2], Lei Zhang [1,2], Penggao Cheng [1,2], Jianping Zhang [1,2] and Na Tang [1,2,*]

[1]Tianjin Key Laboratory of Brine Chemical Engineering and Resource Eco-utilization, College of Chemical Engineering and Material Science, Tianjin University of Science and Technology (TUST), 13$^{th}$Avenue 29, TEDA, Tianjin 300457, China

[2]State Key Laboratory of Biobased Fiber Manufacturing Technology, Tianjin University of Science and Technology, 13$^{th}$ Avenue 29, TEDA, 300457 Tianjin, China

*Corresponding author. E-mail address: tjtangna@tust.edu.cn (Na Tang)

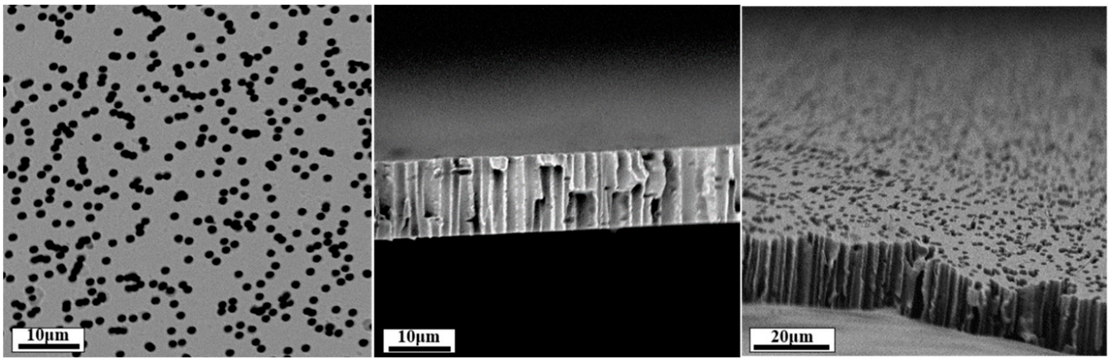


Fig. S1 SEM images of PET nuclear-track membranes.

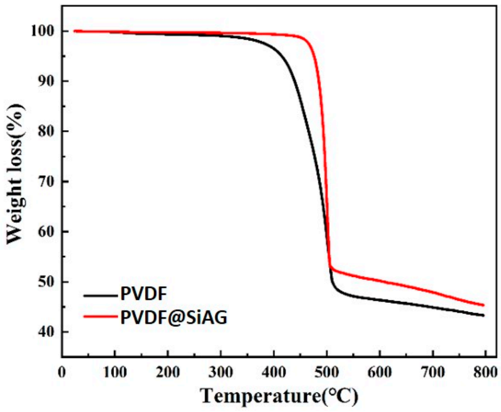


Fig. S2 Thermal gravimetric diagram of the bare PVDF and the PVDF@SiAG.

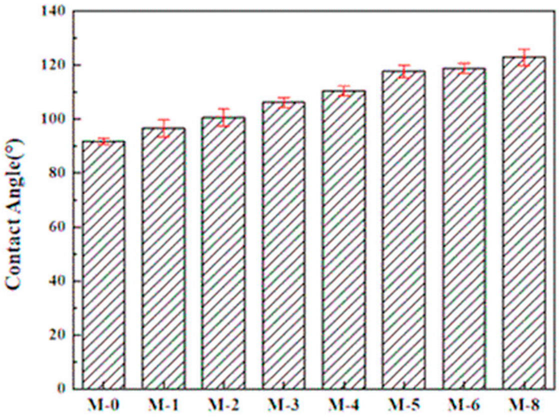

Fig. S3 Dependence of the apparent contact angle on the PVDF@SiAG/PET membranes.
